# Three-dimensional nanoscale analysis of light-dependent organelle changes in *Arabidopsis* mesophyll cells

**DOI:** 10.1093/pnasnexus/pgac225

**Published:** 2022-10-04

**Authors:** Keiko Midorikawa, Ayaka Tateishi, Kiminori Toyooka, Mayuko Sato, Takuto Imai, Yutaka Kodama, Keiji Numata

**Affiliations:** Biomacromoleules Research Team, RIKEN Center for Sustainable Resource Science, 2-1 Hirosawa, Wako, Saitama 351-0198, Japan; Center for Bioscience Research and Education, Utsunomiya University, Tochigi 321-8505, Japan; Biomacromoleules Research Team, RIKEN Center for Sustainable Resource Science, 2-1 Hirosawa, Wako, Saitama 351-0198, Japan; Department of Material Chemistry, Kyoto University, Katsura, Nishikyo, Kyoto 615-8510, Japan; RIKEN Center for Sustainable Resource Science, 1-7-22 Suehiro-cho, Tsurumi-ku Yokohama, Kanagawa 230-0045, Japan; RIKEN Center for Sustainable Resource Science, 1-7-22 Suehiro-cho, Tsurumi-ku Yokohama, Kanagawa 230-0045, Japan; Biomacromoleules Research Team, RIKEN Center for Sustainable Resource Science, 2-1 Hirosawa, Wako, Saitama 351-0198, Japan; Biomacromoleules Research Team, RIKEN Center for Sustainable Resource Science, 2-1 Hirosawa, Wako, Saitama 351-0198, Japan; Center for Bioscience Research and Education, Utsunomiya University, Tochigi 321-8505, Japan; Biomacromoleules Research Team, RIKEN Center for Sustainable Resource Science, 2-1 Hirosawa, Wako, Saitama 351-0198, Japan; Department of Material Chemistry, Kyoto University, Katsura, Nishikyo, Kyoto 615-8510, Japan

**Keywords:** FE-SEM array tomography, chloroplast, peroxisome, mitochondria, *Arabidopsis thaliana*

## Abstract

Different organelles function coordinately in numerous intracellular processes. Photorespiration incidental to photosynthetic carbon fixation is organized across three subcellular compartments: chloroplasts, peroxisomes, and mitochondria. Under light conditions, these three organelles often form a ternary organellar complex in close proximity, suggesting a connection with metabolism during photorespiration. However, due to the heterogeneity of intercellular organelle localization and morphology, organelles' responses to changes in the external environment remain poorly understood. Here, we used array tomography by field emission scanning electron microscopy to image organelles inside the whole plant cell at nanometer resolution, generating a three-dimensional (3D) spatial map of the light-dependent positioning of chloroplasts, peroxisomes, nuclei, and vacuoles. Our results show, in light-treated cells, the volume of peroxisomes increased, and mitochondria were simplified. In addition, the population of free organelles decreased, and the ternary complex centered on chloroplasts increased. Moreover, our results emphasized the expansion of the proximity area rather than the increase in the number of proximity sites interorganelles. All of these phenomena were quantified for the first time on the basis of nanoscale spatial maps. In summary, we provide the first 3D reconstruction of *Arabidopsis* mesophyll cells, together with nanoscale quantified organelle morphology and their positioning via proximity areas, and then evidence of their light-dependent changes.

Significance Statement In light conditions, chloroplasts, peroxisomes, and mitochondria are often located in close proximity to each other, suggesting an association to photorespiratory metabolism. However, the reaction of organelles to changes in the external environment is less understood. Here, we present a three-dimensional (3D) spatial map of the whole plant cell constructed by array tomography with an electron microscope. We quantitatively analyzed intracellular organelle morphology and proximity sites in nanometer resolution. In light-treated cells, the peroxisomes volume increased, mitochondria simplified, and the proximity area between chloroplasts, peroxisomes, and mitochondria expanded. Furthermore, we found that free mitochondria might be involved in the efficient formation of the ternary complex. Our study reports the first nanoscale 3D reconstruction in *Arabidopsis* mesophyll cells and provides new insights into the light-response of cells.

## Introduction

Intracellular changes in response to the surrounding environment are essential for physiological processes *in vivo*. Especially for plants that cannot move by themselves, elevated environmental adaptability at the cellular level is important as a survival strategy. Plant-specific organelles, the unique stress conditions they are exposed to and their responses, their adaptation to the succession of light-dark cycles, and the shuttling of various metabolites all support the existence of plant-specific interaction mechanisms between organelles (1, 2). However, many of these mechanisms are still poorly understood. Intracellular metabolic flux is mediated by multiple organelles. More specifically, the three plant organelles, chloroplasts, peroxisomes, and mitochondria, participate in photorespiration to recover some of the carbon backbones lost via the oxygenation of RubisCO during photosynthesis. Peroxisomes and mitochondria have long been known to localize near chloroplasts, suggesting a link between organellar contact and photorespiratory metabolism (3, 4). A previous study with a femtosecond laser demonstrated light-dependent interaction between peroxisomes and chloroplasts together with morphological changes of peroxisomes (5), suggesting the existence of a mechanism that controls light-dependent dynamics of organelle shape, movement, and interactions. A better understanding of the regulatory mechanisms underlying interorganelle interactions necessitates a comprehensive analytical method, as organelle morphology and interorganelle contacts are heterogeneous within each cell. Confocal microscopy is perhaps the most common experimental approach to exploring contact sites. However, the distance between the membranes of organelles in close proximity ranges from 10  to 80 nm, which is below the best resolution attained by conventional microscopes (2, 6), and thus these approaches cannot attain the resolution required to reconstruct around contact sites accurately. Transmission electron microscopy (TEM) can provide the needed high-resolution and cellular ultrastructure information at contact sites within the cellular context (7, 8), but suffers from a very low throughput and that makes it unsuitable for a global, systematic analysis of the relatively large plant cell.

To overcome these limitations, we turned to array tomography with a field emission scanning electron microscope (FE-SEM). Array tomography using an electron microscope allows the acquisition of a wide field of three-dimensional (3D) views and opens up the possibility of obtaining an accurate quantification of interorganelle contacts (9, 10). Using this technique, we visualized the single whole cell of *Arabidopsis* (*Arabidopsis thaliana*) for the first time and created a spatial map for various organelles. Using *Arabidopsis* mesophyll cells exposed to light or dark conditions, we imaged and reconstructed the 3D morphology of some cellular organelles in plant cells, including chloroplasts, peroxisomes, and mitochondria. These reconstructed 3D images revealed the morphological changes exhibited by individual organelles and unprecedented details of their membrane proximity sites. Here, we quantified light-dependent morphological changes in chloroplasts, peroxisomes, and mitochondria at the nanoscale, and investigated the formation of a light-dependent multiorganelle complex by performing a quantitative spatial analysis of interorganelle membrane proximity regions.

## Results

**Fig. 1.**
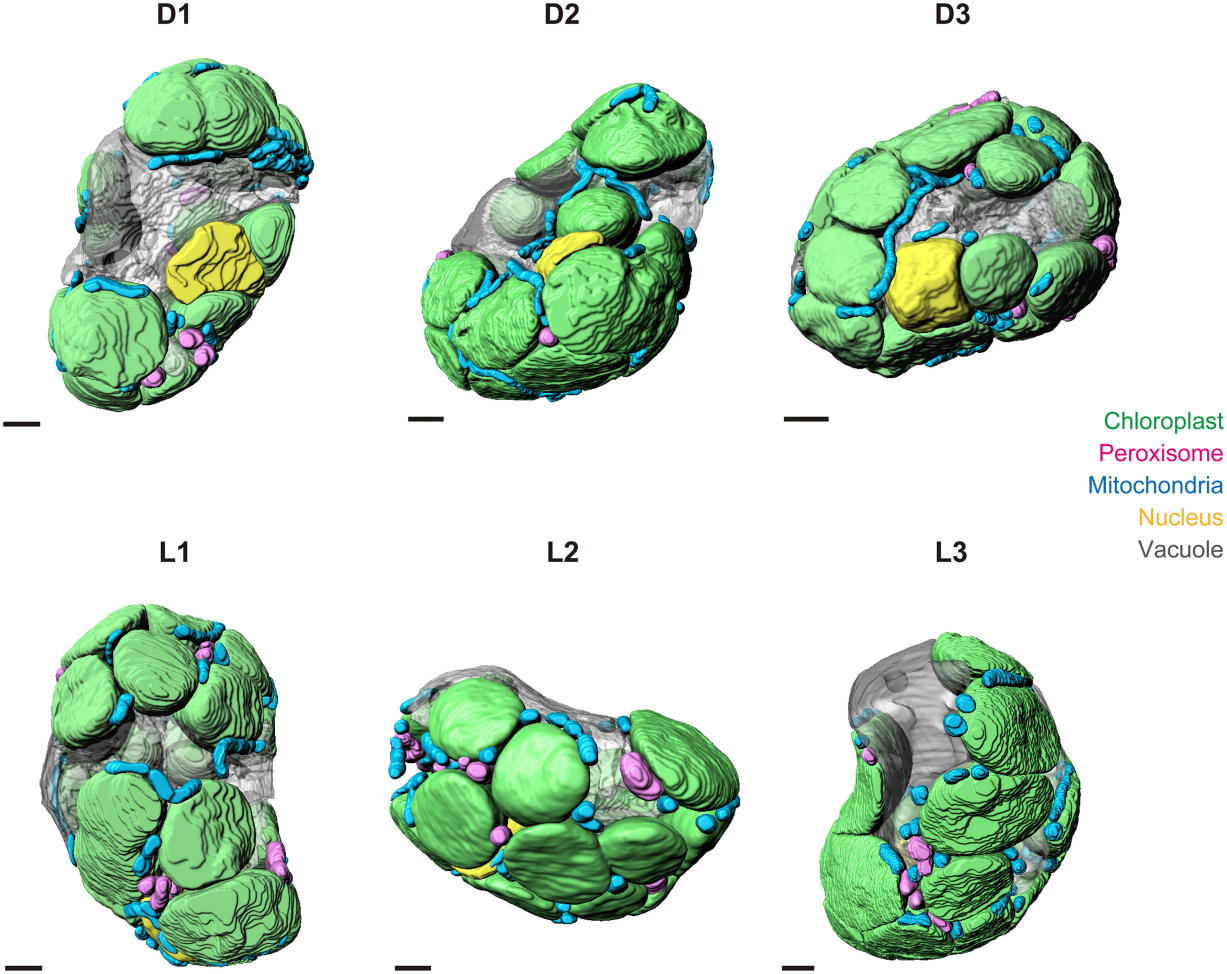
3D reconstruction of whole cells in *Arabidopsis* mesophyll tissue. Three-dimensional images of six plant cells were exposed to dark or light conditions after degassing. The images were constructed from serial section images acquired by FE-SEM. The selected cells belong to the spongy tissue of immature leaves. The position of the cell in the leaf is shown in Figure S2. The cells are arranged so that the top faces the adaxial side and the bottom face the abaxial side. D1 to D3: dark-treated cells; L1 to L3: light-treated cells. Scale bars 2 μm.

The true leaves of 2-week-old *Arabidopsis* seedlings were used as the sample for FE-SEM observation. Since degassing during fixation may affect the condition of organelles, the leaves were degassed prior to pretreatment. The degassed leaves were exposed to white light (30 μmol m^–2^ s^–1^) for 3 h as a pretreatment, and then were incubated for 1 h in either the dark or the light (Fig. S1A). Next, we chemically fixed the leaves with a prefixative solution overnight and then treated them with a postfixative solution in preparation for embedding (Fig. S1A). We produced serial sections with a thickness of 100 nm and deposited them on Kapton tape before electron staining, which consisted of double staining by samarium chloride and lead. We then carbon-coated and scanned the sections with a FE-SEM (Fig. S1B). We selected three cells each from dark-treated and light-treated leaves and created complete 3D images for these six cells (Fig. 1 and Fig. S1B). The leaf cross-sections show unclear differentiation between palisade and spongy tissues; therefore, the selected cells appeared to be immature cells in mesophyll tissue (Fig. S2). Based on the staining patterns obtained, we segmented each two-dimensional (2D) image into five organelles: chloroplasts, peroxisomes, mitochondria, the nucleus, and the vacuole; we then integrated all 2D images into one 3D image per cell (Fig. S1B and Movie S1 to S6). To investigate the remodeling of light-dependent organelle arrangements, we first quantified the basic characteristics of the cell and organelle in dark and light conditions. The cell volume ranged from 1042 to 1711 µm^3^ in the dark-treated group and 1639 to 2518 µm^3^ in the light-treated group. We confirmed that there was no significant difference in cell volume between the two light-treated groups (Table S1). The numbers of the organelles are also shown in Table S1: chloroplast, 15 to 22 (dark) and 17 to 21 (light); peroxisomes, 9 to 13 (dark) and 11 to 16 (light); mitochondria, 34 to 45 (dark) and 36 to 51 (light). These values showed obvious heterogeneity among cells regardless of light treatment. Similarly, the volume of each organelle per cell is shown in Table S1. There was no significant difference between these parameters under the two treatment conditions (Table S1). The intracellular localization of each segmented organelle is shown in Fig. S3. The localization of organelles was spatially restricted by chloroplasts and vacuoles, and most of the peroxisomes and mitochondria appeared to be located between chloroplasts and vacuoles or on the cell surface. This tendency does not seem to be a light-dependent feature. The intracellular proportions of each organelle are shown in Fig. S4. The volumes of chloroplasts and vacuoles were 33% to 47% and 26% to 35% of the cells, respectively, and these two organelles occupied more than 60% of the intracellular space (Fig. S4A). These values were not statistically significant between the two light conditions (Fig. S4A and B). Of the five organelles, we focused on chloroplasts, peroxisomes, and mitochondria, as they participate in photorespiration.

**Fig. 2.**
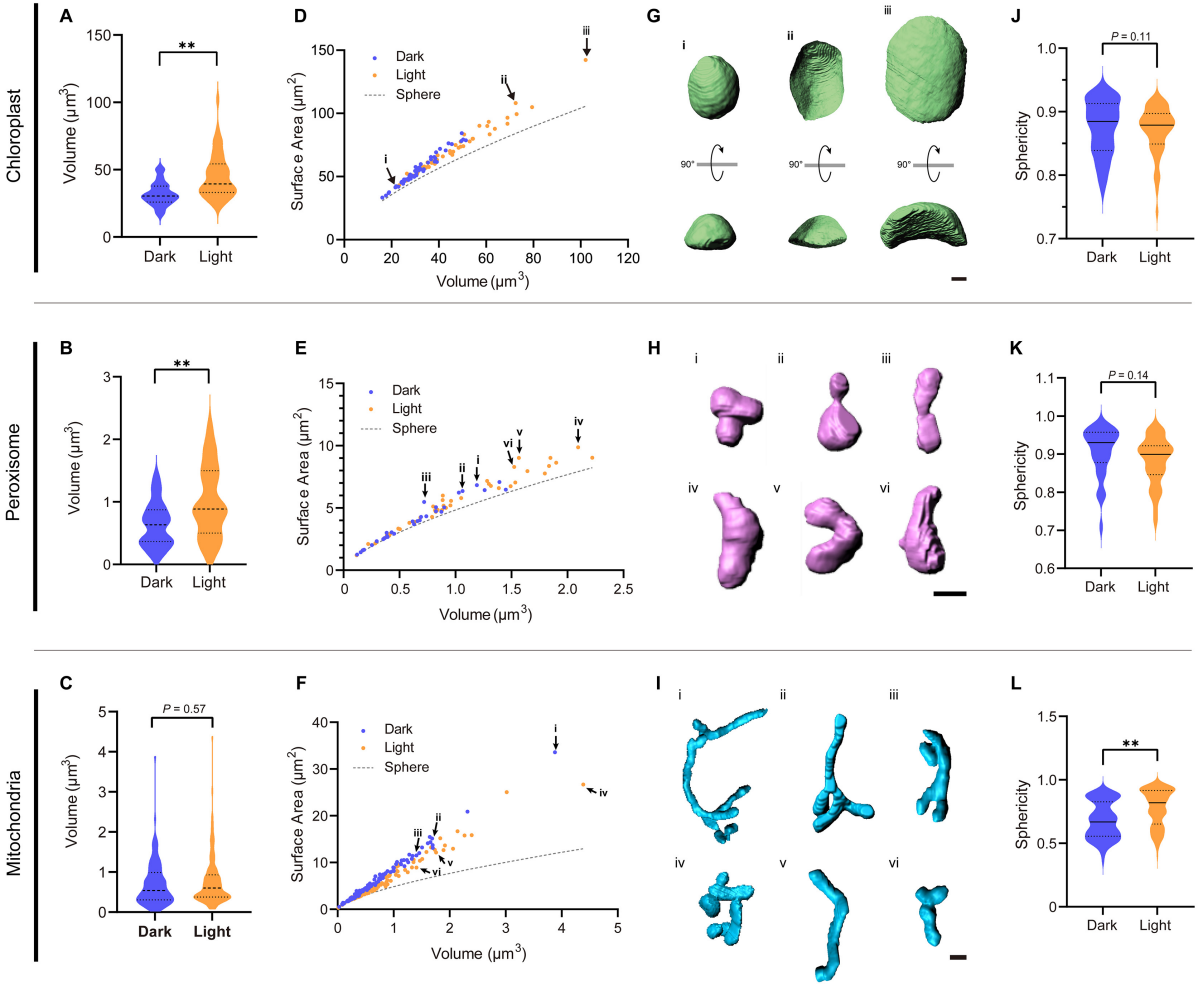
Morphological analysis of chloroplasts, peroxisomes, and mitochondria. All cells were exposed to dark or light conditions after degassing. (A to C) Distribution of organelle volume for chloroplasts (A), peroxisomes (B), and mitochondria (C). (D to F) Relationship between surface area and volume for 109 chloroplasts (D), 73 peroxisomes (E), and 250 mitochondria (F). (G to I) Representative 3D images of three chloroplasts (i and ii, as indicated; D), six peroxisomes (i to vi; E), and six mitochondria (i to vi; F). In each set, dark-adapted organelles are above and light-adapted organelles below. Scale bars, 1.5 µm (G), 1 µm (H, I). (J to L) Sphericity of chloroplasts (J; *n* = 52 [dark], 57 [light]), peroxisomes (K; *n* = 31 [dark], 42 [light]) and mitochondria (L; *n* = 116 [dark], 134 [light]). For (A to C) and (J to L), the median is shown by black dashed lines, and the 25th and 75th percentiles are indicated by the lower and upper dotted lines, respectively. Statistical significance was determined with a Mann–Whitney *U*-test. **P < 0.01.

In all the calculated parameters, the heterogeneity between cells was high, and the light-dependent characteristics of each organelle could not be found in the mean value for each cell. On the other hand, when comparing median values of chloroplasts, peroxisomes, and mitochondria, it was found that light-treated cells had a large volume of chloroplasts and peroxisomes (Fig. 2A to C). On the other hand, when comparing the distribution of cell and each organelle volumes, the median chloroplast volume of each cell was dependent on the cell volume (Fig. S5A). Furthermore, in the correlation analysis between the median cell volume and organelle volume, a high correlation coefficient was found between the median chloroplast and the cell volumes (*r* = 0.93, *P* ** <0.01) (Fig. S5D). Thus, it is likely that chloroplast volume changes are influenced by the cell volume. No significant correlation was found with cell volume in peroxisomes and mitochondria, and the volume change of peroxisomes is considered to be environment-dependent (Fig. S5B, C, E, and F). We confirmed these light-dependent morphological changes by plotting the relationship between surface area and volume and by comparing the results with a theoretical curve for a sphere (Fig. 2D to F). Here, the farther the surface area of an object is from the curve, the more it deviates from the sphere shape. All three organelles showed greater deviation from the theoretical spherical curve (that is, a higher surface-to-volume ratio) with increasing volume. Among the chloroplasts, individuals that are close to a spherical curve (or have a small volume) show a thick shape, but individual chloroplasts that are far from the curve (labeled in Roman numerals in the Fig. 2D) show a flatter shape (Fig. 2D and G and Fig. S6). However, no light-dependent differences in chloroplast shape were detected (Fig. 2J and Fig. S7A and B). Peroxisomes displayed more variation in morphology, with a wide range of volumes under both light and dark conditions (Fig. 2E and H and Fig. S8). The volume of mitochondria did not differ appreciably between light and dark conditions (Fig. 2C), but the mitochondria in dark-treated leaves did deviate more from a spherical shape, even when compared to mitochondria of similar volume from light-exposed leaves (Fig. 2F). Indeed, a comparison of mitochondrion sphericity clearly illustrated their greater complexity in the dark relative to the other two organelles (Fig. 2I and L, and Fig. S9). The 3D reconstruction of mitochondria revealed that they tend to have more elongated mitochondria in the dark and more simplified shapes in the light. (Fig. 2I). In addition, a comparison of the lengths of mitochondria individual clearly shows that the length in the light-treated cells was shorten (Fig. S10A and B). Also, we adopted the mitochondria complex intensity (MCI) to compare complexity, including branching (11). MCI values for 3D analysis are not affected by volume, so mitochondria of the same shape but different volumes are given the same MCI. As a result of comparing the two treatment groups, the MCI value decreased significantly in the light treatment group (Fig. S10C). Scatter plotting with volume and MCI values showed that many individuals in the light-treated cells were less complex (Fig. S10D). Taken together, our results indicate that peroxisomes expand, while mitochondria simplify, in a light-dependent manner.

**Fig. 3.**
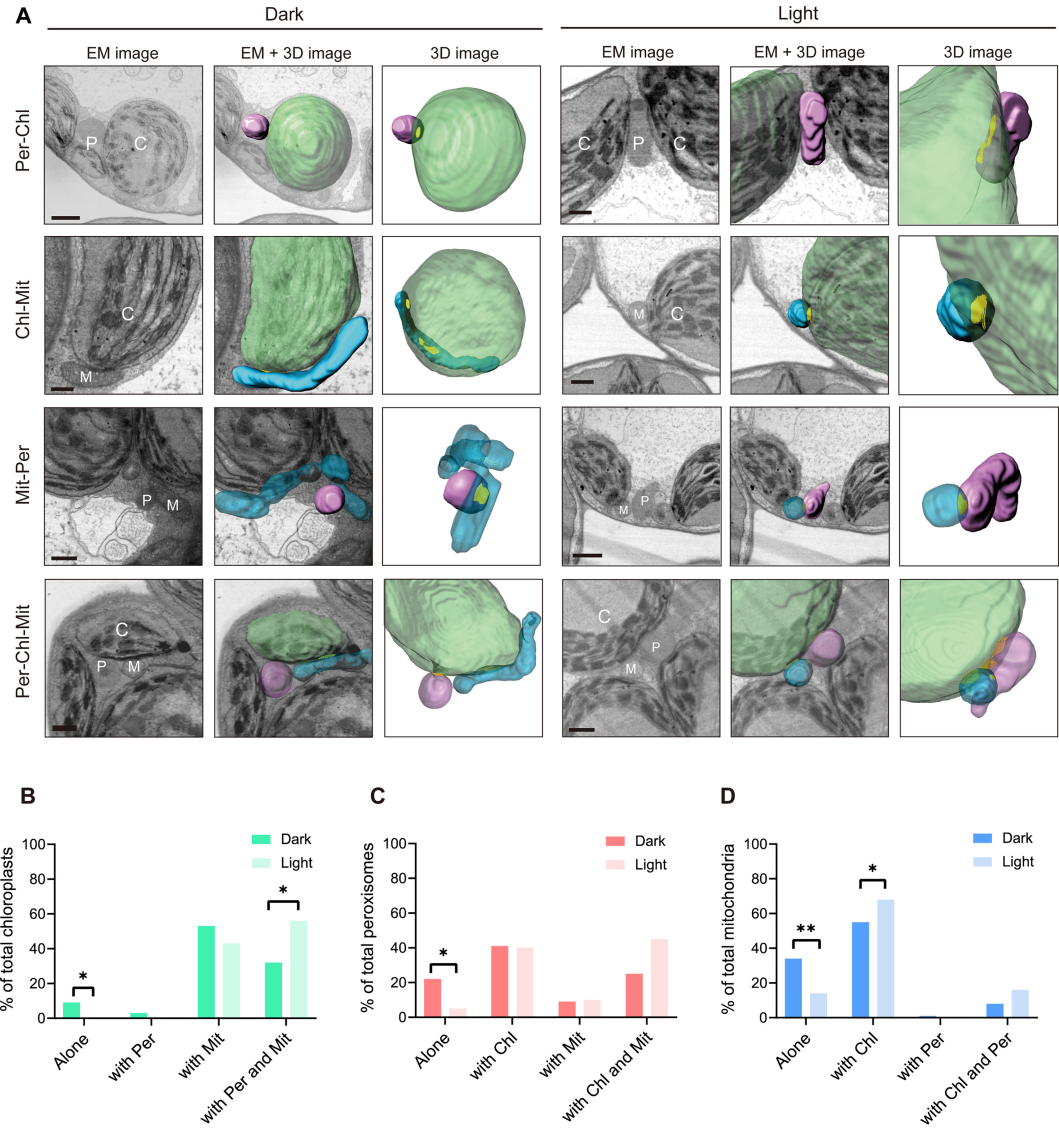
Interorganelle proximity sites between chloroplasts, peroxisomes, and mitochondria. All cells were exposed to dark or light conditions after degassing. (A) Representative 3D and electron micrograph images of interorganelle proximity sites in each light condition. In 3D images, chloroplasts (Chl) are shown in green, peroxisomes (Per) in pink, and mitochondria (Mit) in blue. The interorganelle proximity sites are indicated in yellow. The detection of the interorganelles proximity sites used the algorithm of Imaris software (see Fig. S11). In electron micrograph (EM) images, C, P, and M indicate chloroplasts, peroxisomes, and mitochondria, respectively. Scale bars, 1 μm. (B to D) Percentage of each organelle (B, chloroplasts; C, peroxisomes; D, mitochondria) proximity with other organelles. Statistical significance was determined with a Fisher's exact test,^**^P < 0.01, *P < 0.05.

To investigate how these morphological changes might affect interorganelle proximity, we analyzed how frequently chloroplasts, peroxisomes, and mitochondria came into proximity, and how much of their surface contributed to these interactions (Fig. 3A). Accordingly, we determined the number of interorganelle proximity with a surface–surface contact algorithm provided by the image analysis software Imaris (12). Here, the interorganelle proximity detected the overlap between the shell voxels formed on the surface objects of each organelle constructed by Imaris and the surface voxels of the facing organelles (Fig. 3A and S11). The fractions of each population in proximity were not always symmetric because some individual organelles made simultaneous proximity with two or more organelles of the same type. In cells from dark-treated leaves, 9.6% of chloroplasts did not proximity any mitochondria or peroxisomes (“Alone” in Fig. 3B). In light-exposed cells, all chloroplasts were involved in interactions with at least one other organelle (0% shown as “Alone” in Fig. 3B), proximity to mitochondria (“with Mit”) or forming a ternary complex with mitochondria and peroxisomes (“with Per and Mit”) (Fig. 3B). In the case of peroxisomes, the fraction of free peroxisomes (“Alone” in Fig. 3C) decreased from 22.6% in dark-treated cells to 5.0% in light-exposed cells, while the fraction of peroxisomes engaged in ternary complexes with mitochondria and chloroplasts (“with Mit and Chl”) rose from 25.8% in the dark to 45.0% in the light (Fig. 3C). Free mitochondria (“Alone” in Fig. 3D) decreased from 34.7% in the dark to 14.7% in the light, and this was accompanied by an increase in ternary complexes with chloroplasts and peroxisomes (“with Chl and Per”) from 8.5% in the dark to 16.4% in the light (Fig. 3D). Taking these results together, we concluded that the population of free organelles tends to decrease in light-exposed cells, while ternary organelle complexes tend to increase in frequency. Indeed, Fisher's exact test showed that the distribution of free organelles (alone compared to engaged in contacts with other organelles) in light-exposed cells is significantly different from that observed in dark-treated cells (*P* = 0.027, *P* = 0.038, and *P* = 0.0016, for Fig. 3B to D, respectively). Although the population of mitochondria in a binary complex with chloroplasts increased in the light, from 55.1% to 68.0% (*P* = 0.047), the reciprocal population of chloroplasts engaged in a binary complex with mitochondria tend to decrease under the same conditions, from 53.8% to 43.4% (Fig. 3B). Instead, the population of chloroplasts engaged in a ternary complex increased in the light, from 32.7% to 56.6% (*P* = 0.019) (Fig. 3B). These results suggested that those chloroplasts in binary contact with peroxisomes form a ternary complex with free mitochondria upon light exposure. Therefore, in light-treated cells, free mitochondria may preferentially form a chloroplast-centered ternary complex with peroxisomes, rather than a binary complex.

**Fig. 4.**
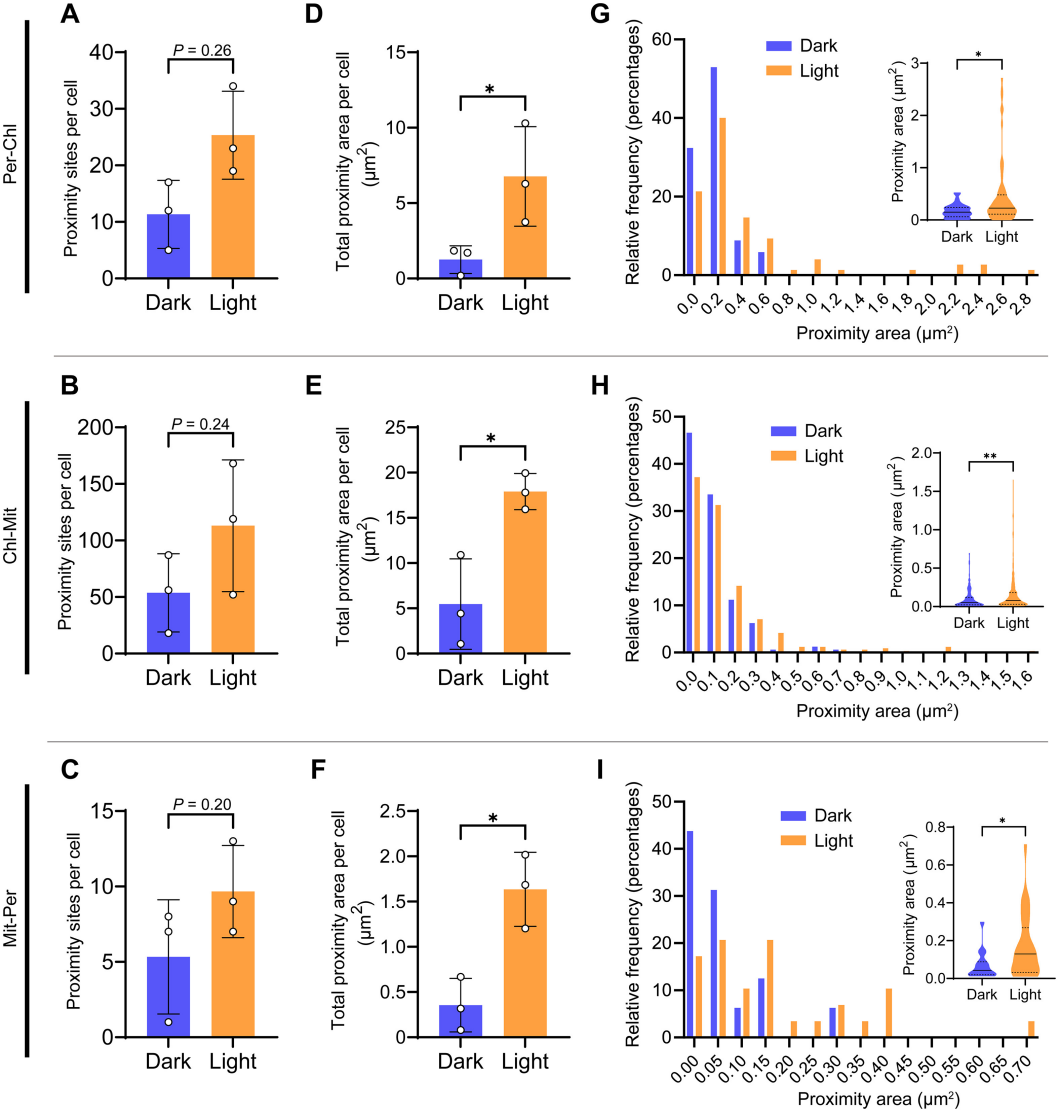
Exposure to light increases the proximity area between organelles. All cells were exposed to dark or light conditions after degassing. (A to C) Total proximity site frequency per cell between light and dark-treated cells between (A) chloroplasts and peroxisomes, (B) chloroplasts and mitochondria, and (C) mitochondria and peroxisomes. (D to F) Total proximity area per cell between light and dark-treated cells between (D) chloroplasts and peroxisomes, (E) chloroplasts and mitochondria, and (F) mitochondria and peroxisomes. Individual data points are shown as open circles and presented as means ± standard deviation (*n* = 3). Statistical significance was determined with a two-tailed unpaired *t*-test. (G to I) Histograms of each proximity area in cells exposed to dark or light conditions. Each bar represents the relative frequency of proximity areas in the dark (blue) or in the light (orange). Insets show all proximity areas. The median is shown by solid black lines, and the 25th and 75th percentiles are shown by lower and upper dotted lines, respectively. Statistical significance was determined with a Mann–Whitney *U*-test. **P < 0.01, *P < 0.05.

Finally, we investigated the proximity frequency and proximity area between the three organelles. The mean proximity sites per cell tended to increase in the light for any proximity type (Per-Chl, Chl-Mit, or Mit-Per), although no significant difference was detected (Fig. 4A to C). Whereas the total surface area involved in interorganelle proximity was approximately three times larger in the light than in the dark (Fig. 4D to F). Very few data points showed a proximity area of more than 0.3 μm^2^ in dark-treated cells (Fig. 4G to I), whereas the area per proximity point between peroxisomes and chloroplasts increased by a median of 66.8% in light-treated cells (Fig. 4G). Similarly, the equivalent area per proximity site between chloroplasts and mitochondria increased by 62.3% and that between mitochondria and peroxisomes rose by 204% (Fig. 4H and I). Since the increase in the proximity area is much more significant than the increase in proximity frequency, we hypothesize that the increase in proximity area per cell is due to the extension from existing proximity sites rather than the newly created proximity sites. We observed a stronger correlation between the volume occupied by peroxisomes and their proximity area with chloroplasts (dark, *r* = 0.81, *P* < 0.0001; light, *r* = 0.45, *P* = 0.0029) than the volume of peroxisomes with the proximity area with mitochondria (dark, *r* = 0.41, *P* = 0.026; light, *r* = 0.27, *P* = 0.085) (Fig. S12A and B). These results suggest that increased peroxisome volume contributes to their greater proximity area with chloroplasts and the possibility that facilitates metabolic flux mediated by hetero organelle complexes.

## Discussion

The results we present herein advance the understanding of light-dependent organelle responses in plant cells. Our results show that the peroxisome volume increases in light-exposed cells, while mitochondria become simplified. While the observed increase in chloroplast volume (or swelling) is cell volume-dependent, we speculate that it also includes the result of the light-induced expansion of the thylakoid lumen (13). Lumenal expansion facilitates the free diffusion of luminal membrane proteins (14). Morphological irregularities associated with increased peroxisome volume and their proximity to other organelles are also supported by analysis using cryo-single-molecule localization microscopy/focused ion-beam SEM　(10). Previous studies showed that the changes in the volume and morphology of an organellar compartment affect the embedded chemical reactions in the cell (15–17). For example, chloroplast volume highly correlates with photosynthetic capacity, with leaf morphology and anatomy playing a critical role in efficiently receiving CO_2_ from intercellular spaces (18, 19). Likewise, changes in the volume of peroxisomes may actively regulate peroxisomal metabolic processes (20), just as morphological changes in mitochondria are highly correlated with cellular energy status (21–25). In previous studies, live-cell imaging observed that mitochondrial morphology became spherical in the dark and elongated in the light (5). Our result is inconsistent with the previous studies; the morphological complexity of mitochondria was increased significantly under the dark condition (Fig. 2L). The difference may be explained due to the method to observe mitochondria. Unlike the previous live-cell imaging study, we observed all mitochondria in the whole cell. Actually, we found some simplified mitochondria in the dark (Figs. S9 and S10). Our data provide new insights into light-dependent mitochondrial morphological changes by 3D analysis of a single whole cell. In contrast to mitochondria, there was no significant difference in the sphericity of chloroplasts and peroxisomes between the light and dark conditions. As the samples were degassed to facilitate immobilization in both conditions, the degas treatment might affect the morphology of chloroplasts and peroxisomes. As in our case, previous reports often used degassing treatment to obtain clear images of the organelle localization and morphology in living plant cells (5, 26–28). To further understand light-dependent morphological changes of chloroplasts and peroxisomes, new technology without the degassing will be needed in the future.

Notably, our results emphasize an increase in proximity area rather than proximity frequency between chloroplasts, peroxisomes, and mitochondria. Previous studies using femtosecond lasers and optical tweezers techniques have shown increased adhesion between peroxisomes and chloroplasts in the light (5, 29). Our findings raise the possibility that the observed increase in adhesive strength might be due to the expansion of the adhesion surface between peroxisomes and chloroplasts we documented here. Expanding already formed proximity areas may be much faster and more efficient than forming new adhesion sites. Increasing these adhesion areas via a change in organelle volume would bypass the need for adenosine triphosphate(ATP) consumption to power organelle movement (30). Membrane contact sites in general coordinate the exchange of metabolites, membrane lipids, and signal transduction molecules (2, 6, 31). Increasing the engaged adhesion surface and/or the number of adhesion foci likely facilitates the distribution of signal transduction substances and metabolites. The changes in organelle morphology and proximity area we established here clearly demonstrated changes in organelle responses due to the light environments. Therefore, the parameters of the organelle proximity site we describe likely reflect the internal and external environment of the cell. Consistent with this hypothesis, many studies have reported alterations in the size and frequency of membrane contact sites related to developmental processes or to biotic or abiotic stress (1, 5, 8, 32–35). The parameters we quantified to compare interactions between organelles, thus characterize the state of cells exposed to light or to darkness, but should be applicable to the dissection of the effects of other growth conditions on organellar morphology and modulation of contact sites. The endoplasmic reticulum (ER) is also essential in considering organelle contact and the effects of the external environment. Capturing contact with ER throughout the cell can be expected to greatly advance a comprehensive understanding of organelle contact (36, 37). Recent tomography analysis has made it possible to acquire high-resolution Z-axis and render hyperfine structures using artificial intelligence technology (38). However, since plant cells are generally much larger than animal cells (the range of diameter is 10 to 100 μm) (39), it is still challenging to apply this technique to plant cells.

Our results quantitatively show on a nanoscale that the volume and morphology of organelles change in response to light exposure, and that the three organelles expand their proximity area to each other. This phenomenon is thought to contribute to photorespiration metabolism through multiple organelles. Direct physical proximity is considered to promote more specific signalling of highly diffusible metabolic intermediates and signalling molecules. According to previous studies, hydrogen peroxide generated from high-light stress is transferred through physical proximity between chloroplasts and the nucleus (40, 41). Related to the organellar proximity, the changes in organellar morphology and volume would also contribute to such metabolic facilitation. This present study showed that peroxisome volume increases in response to light exposure, leading to an expansion of the proximity area with chloroplasts. In addition, it shows the possibility that free mitochondria, simplified by photosynthetic energy supply (23–25), approach chloroplasts and peroxisomes to form a ternary complex, resulting in a new integrated super-compartment optimized for photorespiratory metabolism. Our findings indicate that organelles may adjust their positions along with morphological changes in response to changes in the environment.

## Materials and methods

### Plant materials and growth conditions

*Arabidopsis* (*A. thaliana*; Col-0 accession) seeds were surface sterilized with 70% (v/v) ethanol for 1 min and then with 10% (v/v) NaClO for 15 min, and then washed with sterilized water to remove NaClO. The seeds were sown on half-strength Murashige and Skoog (MS) medium (M5519; Sigma–Aldrich, St. Louis, MO, USA) containing 2.5 mM MES, 1% (w/v) sucrose and 0.8% (w/v) agarose. Seeds were stratified in the dark at 4°C for 2 days and then transferred to constant light (100 μmol m^–2^ s^–1^) at 22°C for germination and growth. For all experiments, 2-week-old seedlings were used.

### Sample preparation for electron microscopy

For electron microscopy analysis, the true leaves of 2-week-old seedlings were used (the long axis is less than 5 mm). The leaves were first placed in a syringe filled with deionized water and gently degassed to facilitate penetration of the fixation fluid (26–28). The degassed leaves were then divided into two groups and placed inside 1.5-mL tubes. Two tubes per condition were preincubated under low light intensity (30 μmol m^–2^ s^–1^) at 22°C for 3 h. Subsequently, the samples from the light-treated group were maintained at 22°C for another hour under the light condition. The dark-treated samples were shielded from light with aluminium foil and kept at 22°C for 1 h.

After light or dark incubation, leaves were fixed for electron microscopy. The samples were submerged in prefixation solution (4% [w/v] paraformaldehyde, 2% [v/v] glutaraldehyde, and 0.05 M cacodylic acid buffer), allowed to stand at room temperature for 30 min, and fixed on ice overnight. To avoid undesired organelle alterations during chemical fixation, samples were maintained in the same light and dark conditions experienced during treatments. The fixed samples were washed six times (10 min each) with 0.05  M sodium cacodylate buffer (pH 7.4), submerged in another fixative (1% [w/v] OsO_4_, 0.05 M cacodylic acid buffer) and allowed to stand at room temperature for 3 h. After fixation, the samples were washed three times with ultrapure water (5 min each). Subsequently, the samples were dehydrated in a gradient methanol series from 25%, 50%, 75%, 99%, and 2 × 100% methanol (v/v) and embedded in Epon 812 resin (TAAB, UK).

### Field emission scanning electron microscopy

For serial sections, thin-layer sections with a thickness of 100 nm were generated with a diamond knife (DIATOME histo, Diatome, Switzerland) with an automatic tape collection ultramicrotome ATUMtome (RMC-Boeckele, AZ) and collected on Kapton tape (42). The sections on Kapton tape were then attached to carbon tape on one glass slide and stained for 30 min with 4% (w/v) samarium chloride for 30 min and 5 min with Reynold's lead citrate (34) at room temperature. After being washed and dried, the samples were carbon-coated using a vacuum film deposition system (JEE-420, JEOL, Japan). The slides were observed with FE-SEM (ZEISS Gemini SEM300, Carl Zeiss, Germany) equipped with a backscattered electron detector (BSD4) at 3.8 kV.

### 3D Reconstruction and image analysis

From the rough images collected for all sections, three cells were selected from each dark- or light-treated sample (dark-treated group: D1, D2, and D3 cells; light-treated group: L1, L2, and L3 cells). Cells of similar size and located in the middle layer of the mesophyll were selected (Fig. S2). Each cell was then scanned in more detail. The resulting serial images were saved as tiff files after adjusting the alignment. Segmentation was performed using Imaris software (v8.4.1, Bitplane) to create a 3D model of the cells of interest from stacks of tiff files. After loading the serial images into Imaris, the electronic images were inverted and adjusted for contrast. For structures to be reconstructed in 3D, namely chloroplasts, peroxisomes, mitochondria, nuclei, and vacuoles, region selection was performed manually, and a 3D rendering of each organelle was generated using the “Surfaces” tool in Imaris.

For morphological analyses, volumes and surface areas were measured by Imaris software. For surface proximity analyses, the rendered surfaces were used to determine the extent of proximity between the surface of different organelles using the surface–surface contact area algorithm loaded into Imaris (12) (Fig. S11B). This algorithm was reported to detect the surface contact area between two surfaces (12) by creating a voxel shell around the primary surface created by the “Surfaces” tool and calculating the fraction of that shell that overlaps the secondary surface (Fig. S11B). This surface area is estimated by taking the number of overlapping voxels and multiplying it by the area of a single (XY pixels). In our case, the XY pixels are calculated as 10 × 10 nm, and the part that is in close proximity to the membrane within a single voxel (10 × 10 × 100 nm) as the membrane proximity site. The same calculation was performed on the six cells (L1, L2, L3, D1, D2, and D3 cells). These surfaces were also used to determine proximity frequency. Organelles with proximity areas were counted as individuals in proximity with different organelles.

### Statistical analyses

All data were plotted using GraphPad Prism 9.2.0 (GraphPad Software Inc.). Statistical analyses of the volume, sphericity, motility, and proximity areas were performed using a nonparametric Mann–Whitney *U*-test (Fig. 2A to C and J, Fig. 4G to I). Statistical analyses of proximity area and frequency per cell were conducted using an unpaired *t-*test with Welch's correction (Fig. 4A to F). For proximity frequency analysis, a Fisher's exact test was used to determine the significance for percentage of each organelle in proximity with other organelles (Fig. 3B to D). *P*-values of < 0.05 were considered significant.

## Supplementary Material

pgac225_Supplemental_Files

## Data Availability

All data needed to evaluate the conclusions in the paper are present in the paper and/or the Supplementary Materials.
