# Supplementary material for: Three-dimensional nanoscale analysis of light-dependent organelle changes in Arabidopsis mesophyll cells
Source: PNAS Nexus. 2022 Oct 4;1(5):pgac225. doi: 10.1093/pnasnexus/pgac225 (PMC9802074; doi:10.1093/pnasnexus/pgac225)
Supplement: pgac225_Supplemental_Files [file pgac225_supplemental_files.zip › PNASNEXUS-PNASNEXUS-2022-00152-s01.pdf]

PNAS nexus

Supplementary Information for

Three-dimensional nanoscale analysis of light-dependent organelle changes in *Arabidopsis* mesophyll cells

Keiko Midorikawa, Ayaka Tateishi, Kiminori Toyooka, Mayuko Sato, Takuto Imai, Yutaka Kodama*, Keiji Numata*

*Correspondence to: Yutaka Kodama and Keiji Numata
Email: kodama@cc.utsunomiya-u.ac.jp (Y. K.), keiji.numata@riken.jp (K. N.)

This PDF file includes:

Figures S1 to S12
Tables S1
Legends for Movies S1 to S6

Other supplementary materials for this manuscript include the following:

Movies S1 to S6

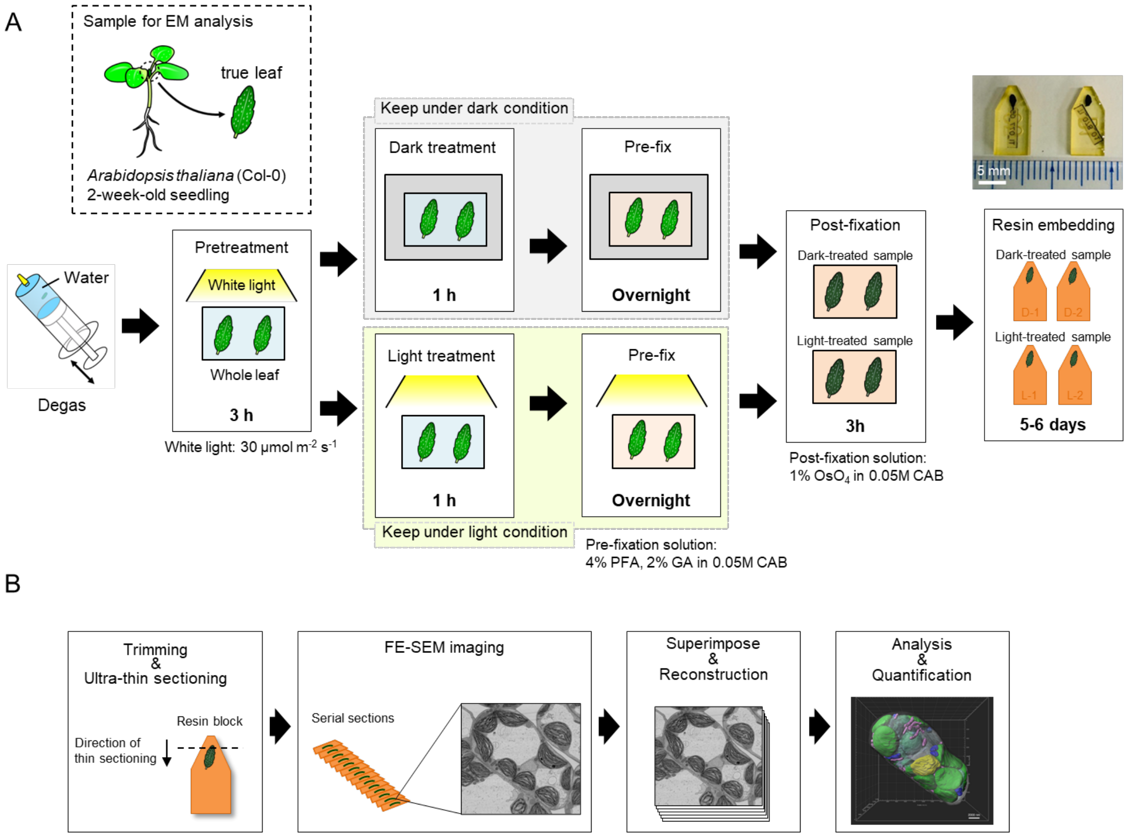

Fig. S1. Experimental workflow. (A) Light and dark treatments and fixation method. For electron microscopy analysis, the true leaves of 2-week-old seedlings were used. Arabidopsis leaves were exposed to light for 3 h for pre-treatment and then transferred to their respective light conditions until post-fixation. All leaves were degassed before being exposed to light or dark treatment. (B) Array tomography method. Serial sections were prepared from resin-embedded samples, from which images were acquired by FE-SEM. Analysis was performed based on the 3D image obtained from the serial section images.

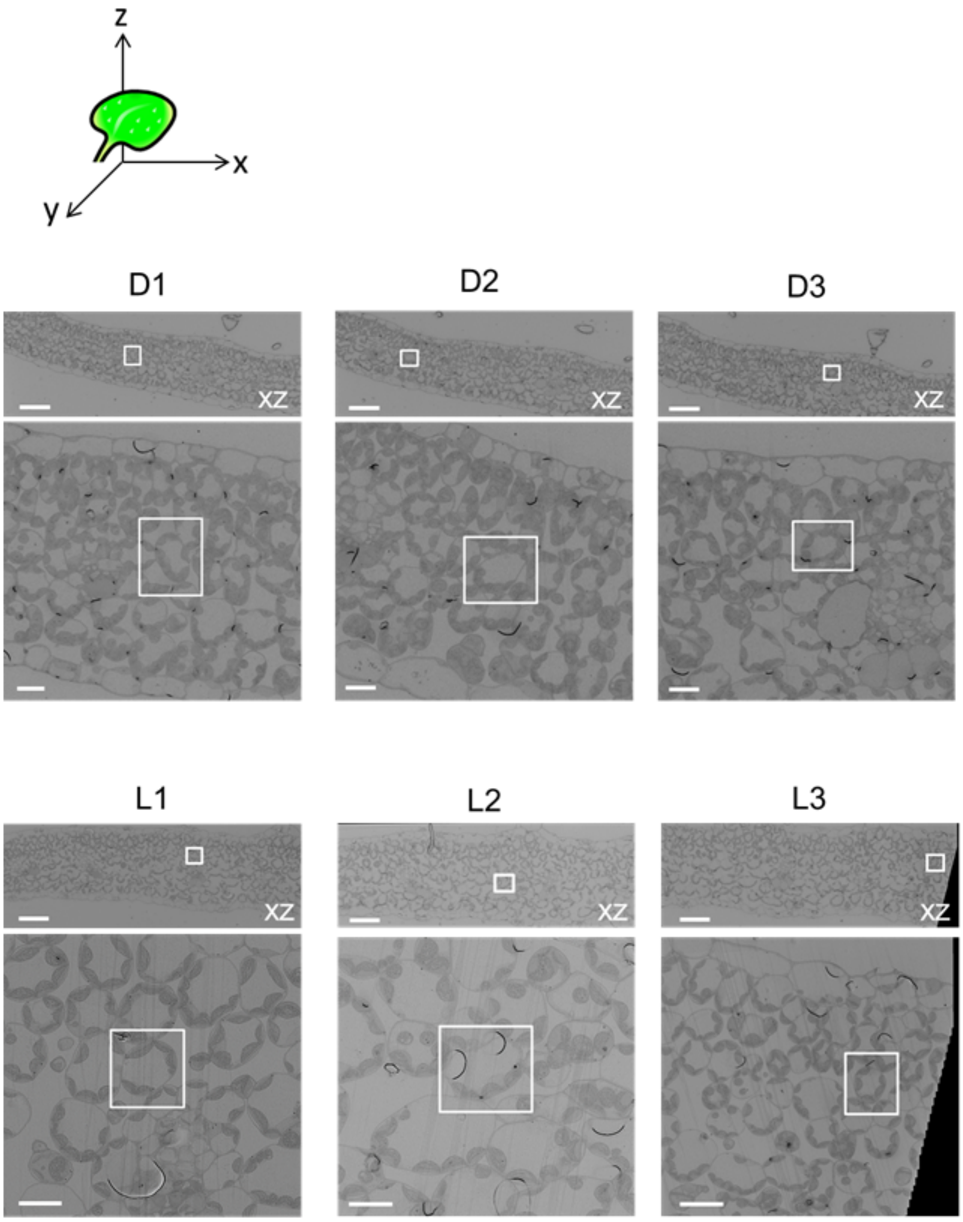

Fig. S2. Location of selected immature cells of mesophyll tissue in Arabidopsis leaf. The cells surrounded with rectangles represent selected cells. The images show the XZ cross-section of the leaf (upper left illustration). The bottom column is a magnified image of the area around a particular cell in the upper column. D1-D3: dark-treated cells, L1-L3: light-treated cells. The scale bars indicate 50 μm (upper column) and 10 μm (bottom column), respectively. All cells were exposed to dark or light conditions after degassing.

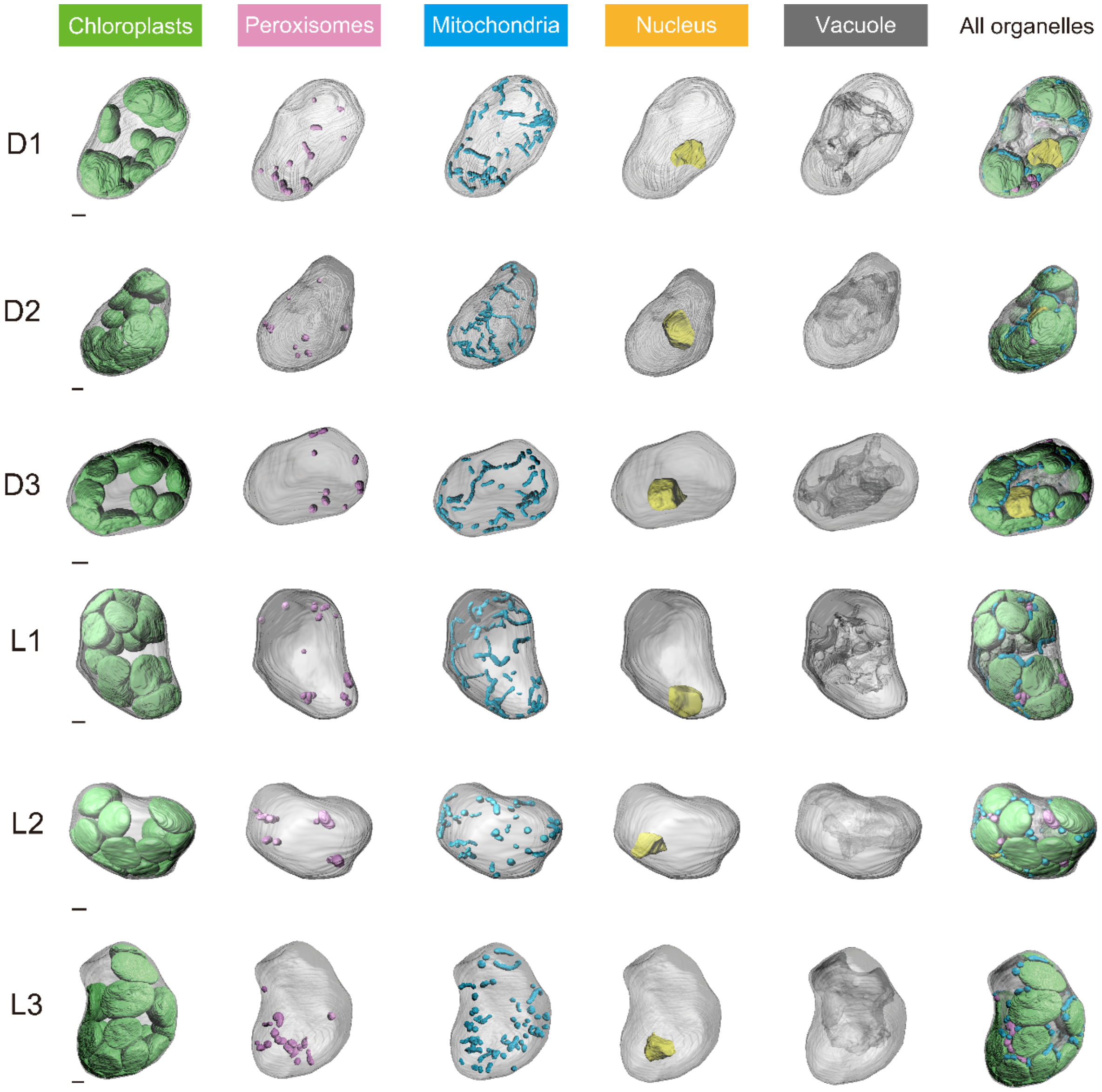

Fig. S3. 3D renderings of all mesophyll cells and organelles analyzed in this study. Colored-coded is chloroplasts, peroxisomes, mitochondria, nuclei, and vacuoles. Scale bars indicate 2 μm respectively. D1–D3, dark-treated cells; L1–L3, light-treated cells.

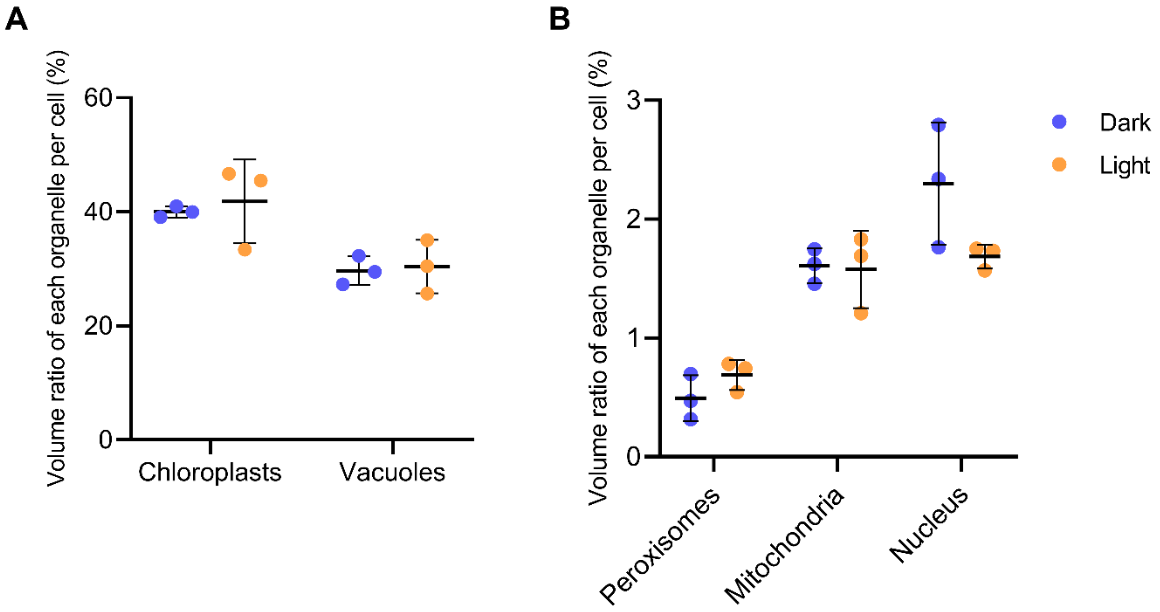

Fig. S4. Relative contribution of each segmented organelle to total cell volume for all analyzed cells. Each circle represents the volume ratio of each organelle per cell. Error bars indicate mean value ± SD. (A) Chloroplasts and vacuoles, (B) Peroxisomes, mitochondria, and nucleus. D1-D3, dark-treated cells; L1-L3, light-treated cells.

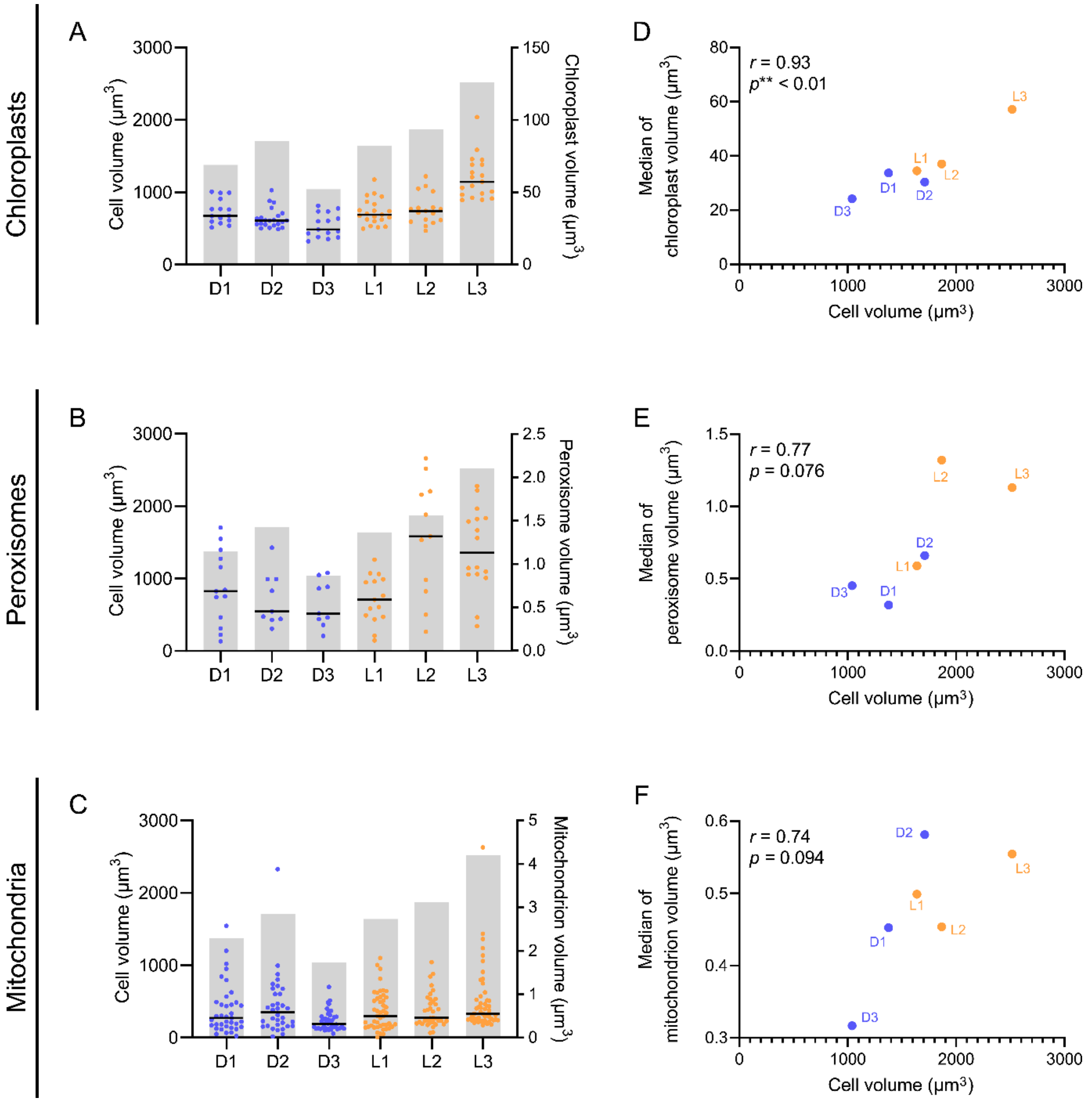

Fig. S5. Comparison of organelle morphology and cell volume. Each circle represents a single organelle. (A-C) Comparison of volume distribution of each organelle per cell and cell volume. The black lines show the median value of each organelle's volume. The gray bar graph shows the cell volume. (D-F) Correlation analysis of the median value and cell volume. *r* represents Pearson's correlation coefficient. D1–D3, dark-treated cells; L1–L3, light-treated cells.

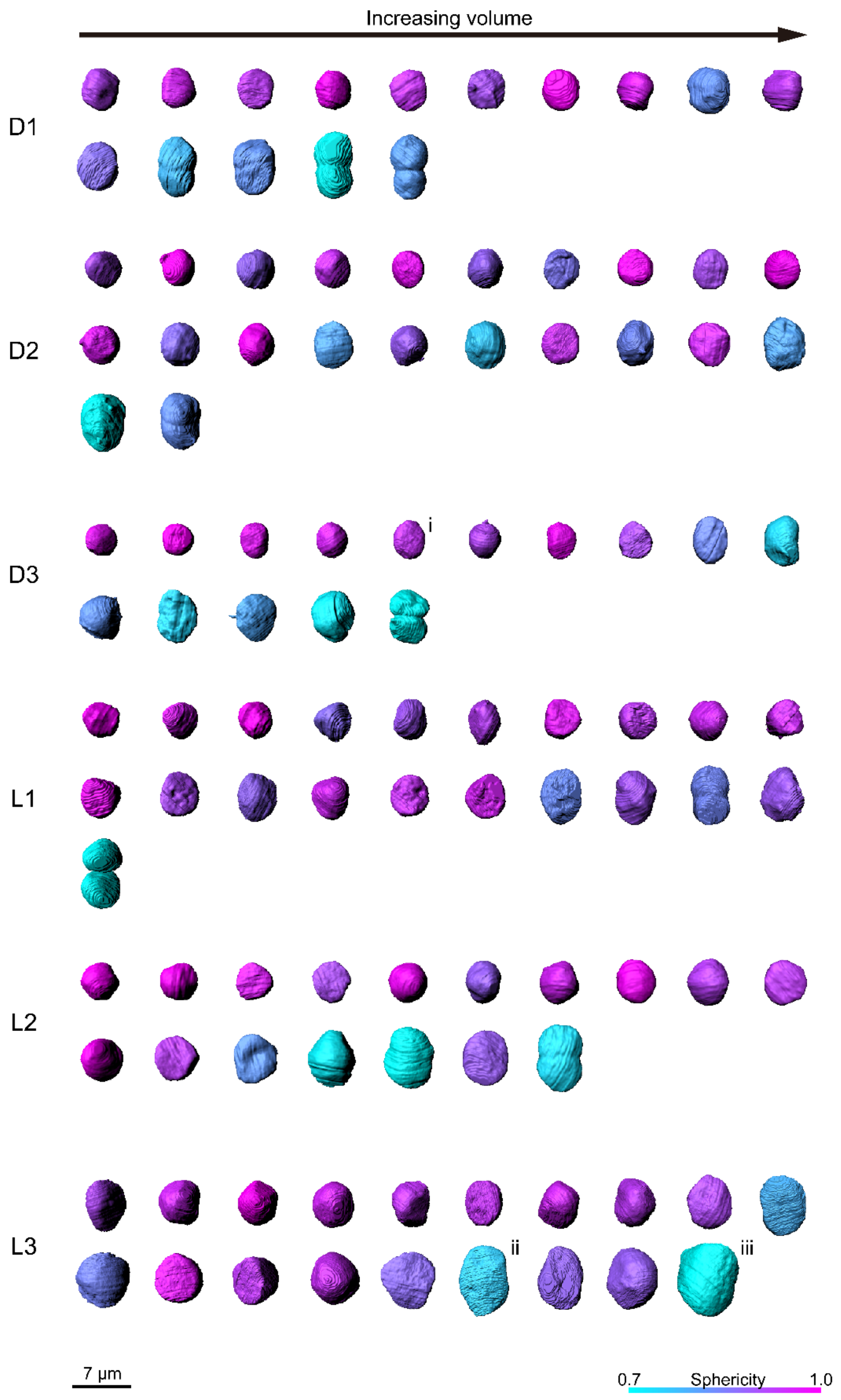

Fig. S6. FE-SEM segmentation of chloroplasts. Chloroplasts are ordered from top to bottom based on their volume, and color-coded according to their degree of deviation from a spherical shape (cyan: less spherical; magenta: more spherical). D1-D3: dark-treated cells, L1-L3: light-treated cells. Roman numerals correspond to the mitochondria shown in Fig. 2D and G. All cells were exposed to dark or light conditions after degassing.

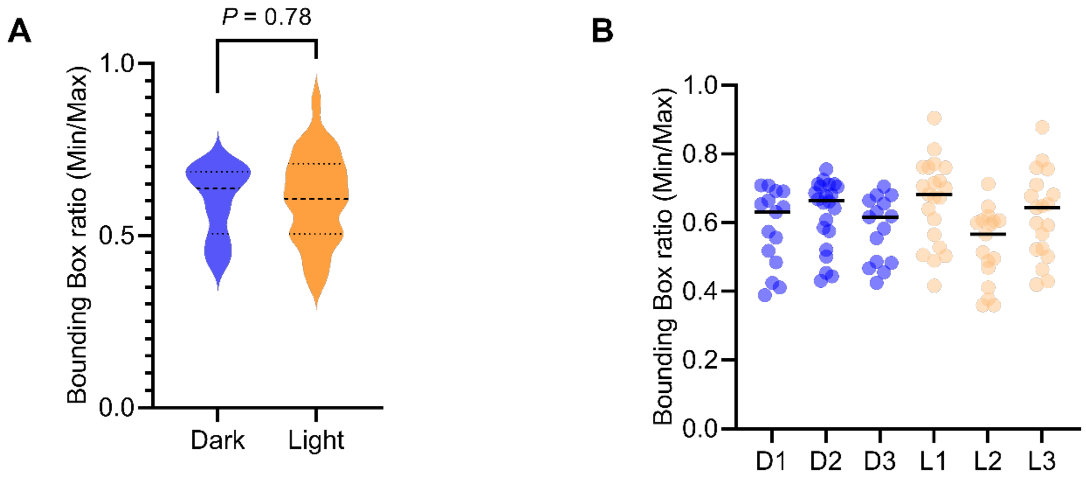

Fig. S7. Morphological analysis of chloroplasts. (A) The violin plots show the ratio of the bounding box. The median is shown by black dashed lines, and the 25th and 75th percentiles are indicated by the lower and upper dotted lines, respectively. Statistical significance was determined with a Mann-Whitney *U* test. *P*-value of < 0.05 was considered significant. (B) Dot plot showing the bounding box ratio of chloroplasts per cell. D1-D3: dark-treated cells, L1-L3: light-treated cells. All cells were exposed to dark or light conditions after degassing.

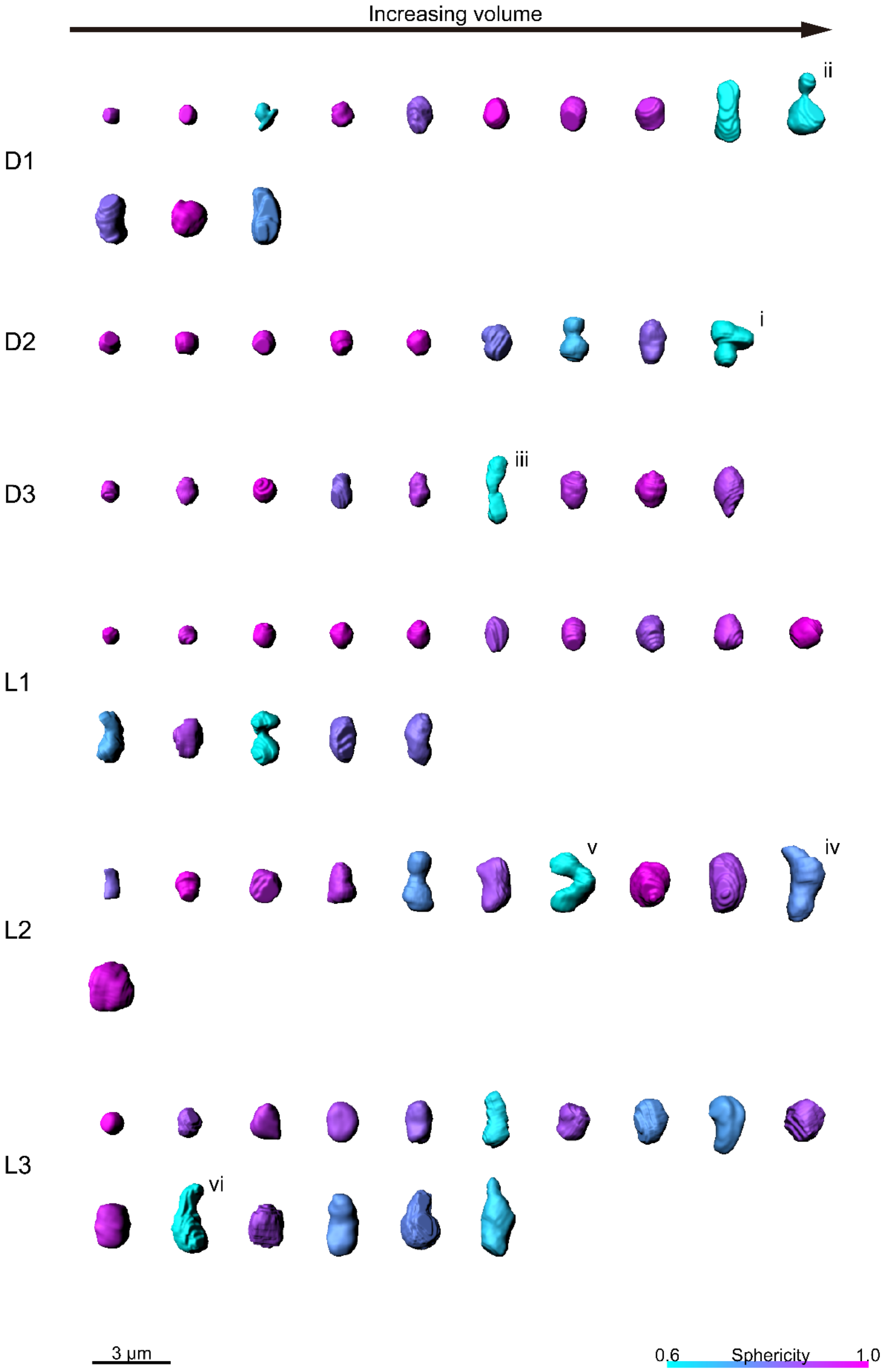

Fig. S8. FE-SEM segmentation of peroxisomes. Peroxisomes are ordered from top to bottom based on their volume, and color-coded according to their degree of deviation from a sphere (cyan: less spherical; magenta: more spherical). Top: dark-treated cells; bottom: light-treated cells. Roman numerals correspond to the peroxisomes shown in Fig. 2E and H. All cells were exposed to dark or light conditions after degassing.

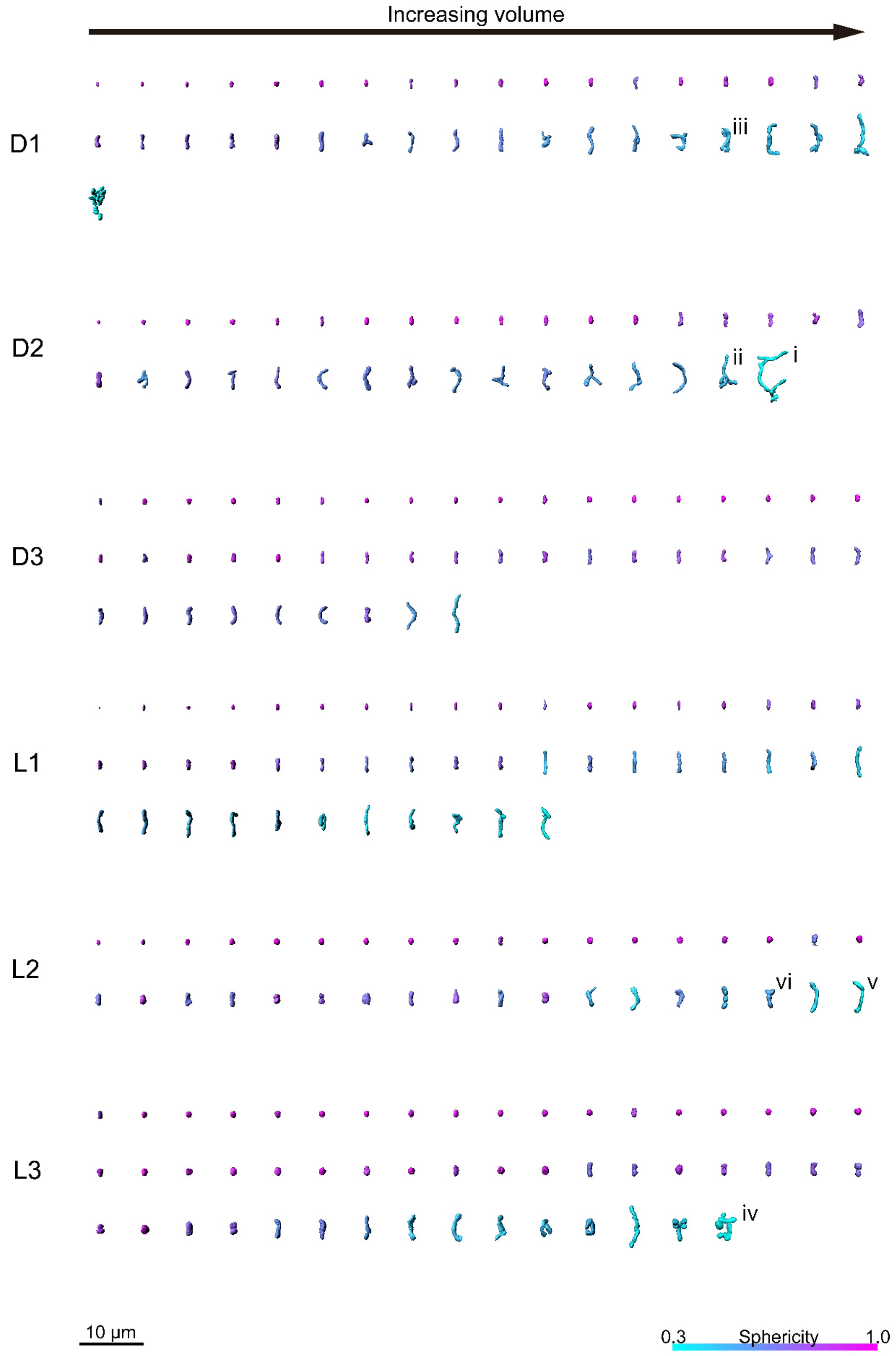

Fig. S9. FE-SEM segmentation of mitochondria. Mitochondria are ordered from top to bottom based on their volume, and color-coded according to their degree of deviation from a spherical shape (cyan: less spherical; magenta: more spherical). Top: dark-treated cells; bottom: light-treated cells. Roman numerals correspond to the mitochondria shown in Fig. 2F and I. All cells were exposed to dark or light conditions after degassing.

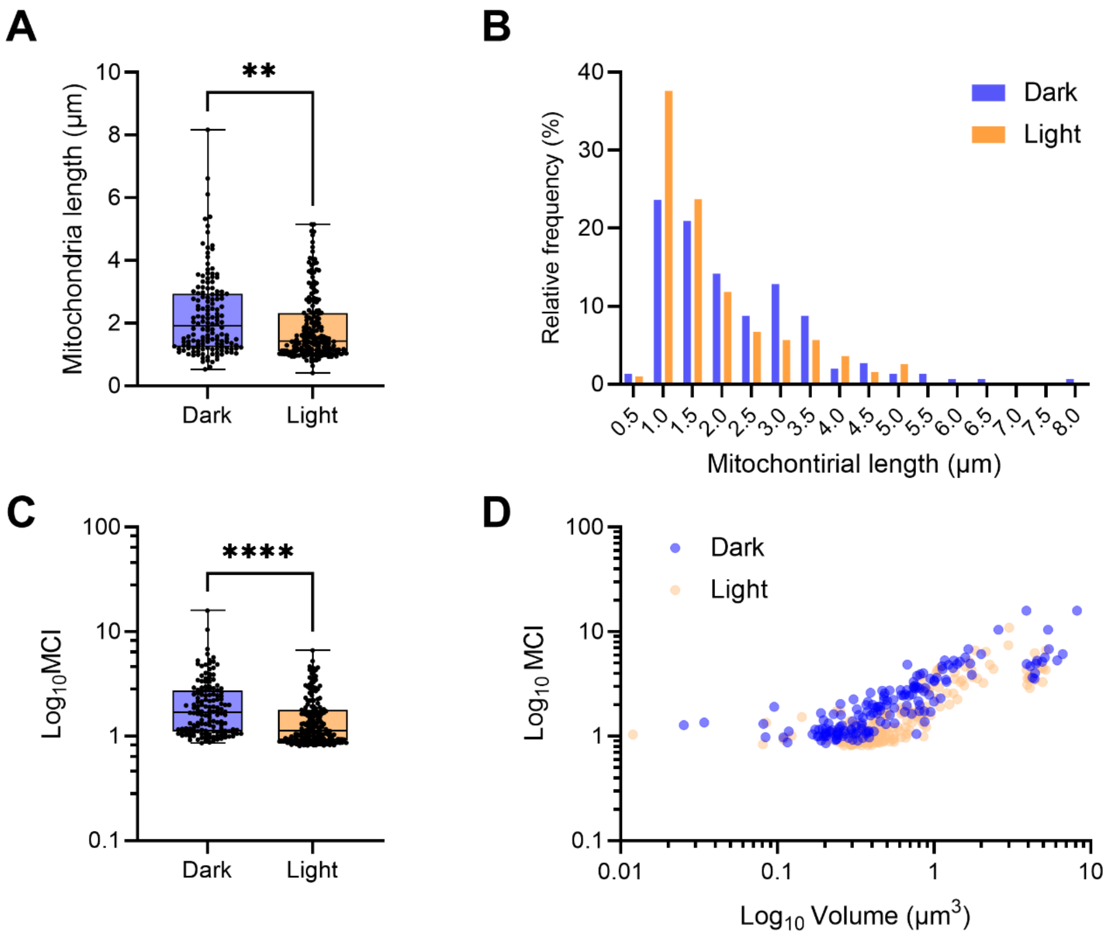

Fig. S10. Morphological analysis of mitochondria. (A) The distribution for length mitochondrion length is shown as box plots for n = 116, 134. (B) Mitochondria length histograms in dark- (blue) and light- (orange) treated cells. (C) Distribution of mitochondria complex intensity (MCI) in each mitochondrion. (D) Bivariate plot of volume and MCI for grown under the dark and light samples. Each point represents a single mitochondrion. The bars indicate the median value. Statistical significance was determined with a Mann-Whitney *U* test. Median is a thick black line, and the 25th and 75th percentiles are dotted lines. $^{**}P < 0.01$, $^{****}P < 0.0001$.

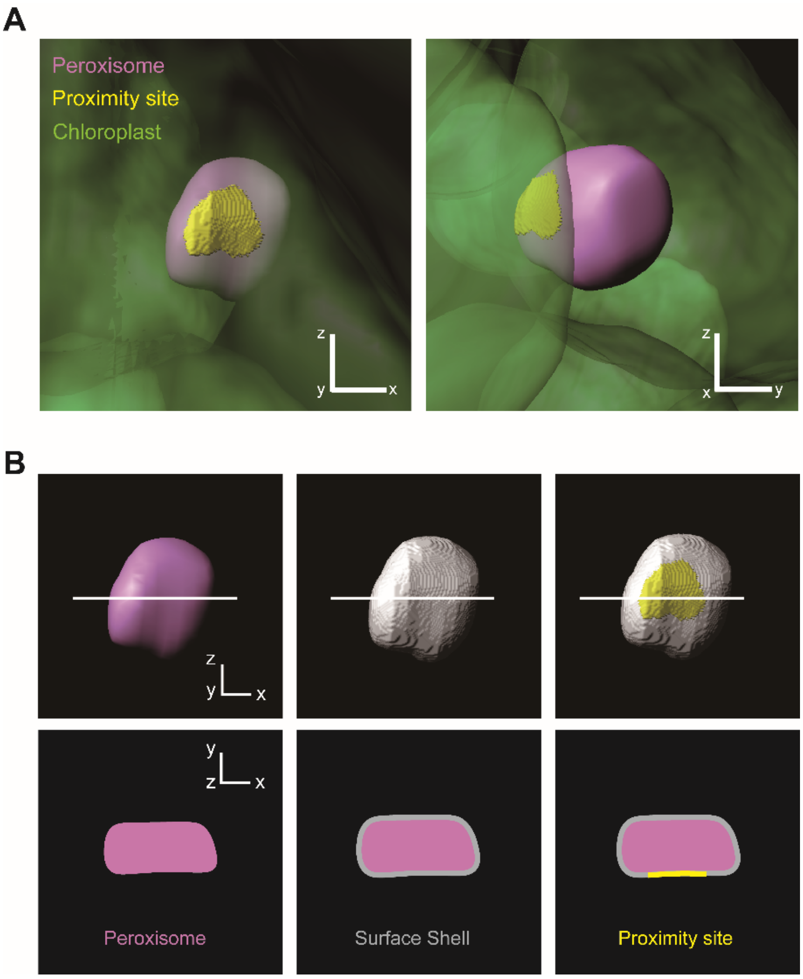

Fig. S11. Method of surface proximity analysis. (A) The proximity site (yellow) between peroxisome (pink) and chloroplast (green) was determined by a surface-surface contact algorithm provided by Imaris. (B) The analysis example of the proximity site (yellow). The surface shell 1 voxel thick (10 nm × 10 nm × 100 nm) (gray) was identified that covered the surface of the peroxisome (pink). Any surface shell voxel that overlapped with the chloroplast surface was designated as the proximity site, shown as yellow in B. The XYZ coordinate plane in the corner of A and B indicates orientation.

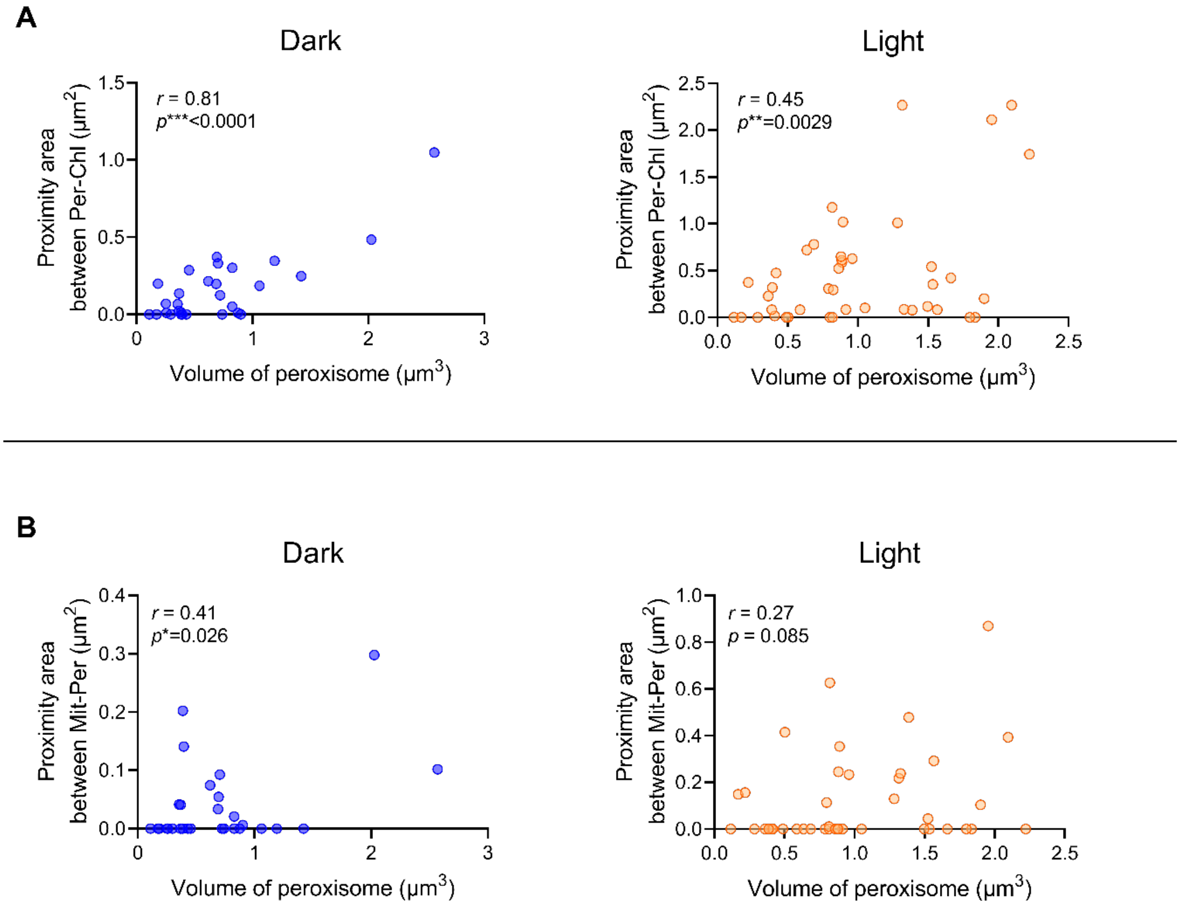

Fig. S12. Correlation analysis between organelle volume and proximity area. Each circle represents a single organelle**.** (A) Peroxisome-chloroplast proximity areas as a function of peroxisome volume. (B) Volume of peroxisomes vs. peroxisome-mitochondria proximity areas as a function of peroxisome volume. *r* represents the Pearson's correlation coefficient. Left, dark-treated cells; right: light-treated cells. All cells were exposed to dark or light conditions after degassing.

Table S1. Organelle measurement in all analyzed cells. The statistical analysis for the mean of three cells under each light condition was according to a two-tailed Welch's t-test.

	Dark			Light			Mean ± SD		
	**D1**	**D2**	**D3**	**L1**	**L2**	**L3**	**Dark**	**Light**	**p-Value**
Cell volume (μm^3)	1,377	1,711	1,042	1,639	1,868	2,518	1,377 ± 334.8	2,008 ± 455.9	0.1316
Cell surface area (μm^2)	678.1	784.9	548.4	754.3	807.5	987.6	670.6 ± 118.4	849.8 ± 122.3	0.1422
Chloroplast number	15	22	15	21	17	19	17 ± 3	19 ± 2	0.5687
Chloroplast total volume (μm^3)	550.5	701.3	406.8	738.5	652.1	1,145.0	552.9 ± 147.0	845.2 ± 263.2	0.1877
Chloroplast total surface area (μm^2)	924.8	1,214.6	753.3	1,294.5	1,071.5	1,648.5	964.2 ± 233.1	1,338.0 ± 291.0	0.1608
Peroxisome number	13	9	9	15	11	16	10 ± 2	14 ± 3	0.1461
Peroxisome total volume (μm^3)	9.5	5.4	4.9	8.9	14.6	18.7	6.6 ± 2.5	14 ± 4.9	0.1019
Peroxisome total surface area (μm^2)	55.2	33.6	32.1	56.2	72.1	97.3	40.3 ± 12.9	75.2 ± 20.7	0.0808
Mitochondrion number	37	34	45	47	36	51	39 ± 6	45 ± 8	0.3462
Mitochondrion total volume (μm^3)	20.4	23.4	36.7	28.9	23.4	42.9	26.8 ± 8.7	31.7 ± 10.0	0.5582
Mitochondrion total surface area (μm^2)	173.8	203.3	330.1	222.4	178.7	262.4	235.7 ± 83.0	221.2 ± 41.9	0.8040
Nucleus volume (μm^3)	32.2	30.2	29.1	28.7	32.4	39.5	30.5 ± 1.6	33.6 ± 5.5	0.4426
Nucleus surface area (μm^2)	64.3	69.3	54.3	63.2	71.4	71.1	62.6 ± 7.6	68.6 ± 4.6	0.4311
Vacuole volume (μm^3)	405.5	552.2	284.3	420.8	568.8	881.9	413.7 ± 134.2	624.0 ± 235.4	0.2677
Vacuole surface area (μm^2)	460.8	505.8	456.4	498.7	588.1	657.4	474.3 ± 27.3	581.4 ± 79.5	0.1339

Movie S1 (separate file). 3D reconstructed image of a dark-treated whole cell (D1). The image shows an immature mesophyll cell in the Arabidopsis leaf. Segmentation and 3D rendering were performed by Imaris software (v8.4.1, Bitplane). Chloroplasts (green), peroxisomes (pink), mitochondria (blue), nuclei (yellow) and vacuoles (gray) were reconstructed. After degassing, the cells were retained in the dark treatment.

Movie S2 (separate file). 3D reconstructed image of a dark-treated whole cell (D2). The image shows an immature mesophyll cell in the Arabidopsis leaf. Segmentation and 3D rendering were performed by Imaris software (v8.4.1, Bitplane). Chloroplasts (green), peroxisomes (pink), mitochondria (blue), nuclei (yellow) and vacuoles (gray) were reconstructed. After degassing, the cells were retained in the dark treatment.

Movie S3 (separate file). 3D reconstructed image of a dark-treated whole cell (D3). The image shows an immature mesophyll cell in the Arabidopsis leaf. Segmentation and 3D rendering were performed by Imaris software (v8.4.1, Bitplane). Chloroplasts (green), peroxisomes (pink), mitochondria (blue), nuclei (yellow) and vacuoles (gray) were reconstructed. After degassing, the cells were retained in the dark treatment.

Movie S4 (separate file). 3D reconstructed image of a light-treated whole cell (L1). The image shows an immature mesophyll cell in the Arabidopsis leaf. Segmentation and 3D rendering were performed by Imaris software (v8.4.1, Bitplane). Chloroplasts (green), peroxisomes (pink), mitochondria (blue), nuclei (yellow) and vacuoles (gray) were reconstructed. After degassing, the cells were exposed to the light treatment.

Movie S5 (separate file). 3D reconstructed image of a light-treated whole cell (L2). The image shows an immature mesophyll cell in the Arabidopsis leaf. Segmentation and 3D rendering were performed by Imaris software (v8.4.1, Bitplane). Chloroplasts (green), peroxisomes (pink), mitochondria (blue), nuclei (yellow) and vacuoles (gray) were reconstructed. After degassing, the cells were exposed to the light treatment.

Movie S6 (separate file). 3D reconstructed image of a light-treated whole cell (L3). The image shows an immature mesophyll cell in the Arabidopsis leaf. Segmentation and 3D rendering were performed by Imaris software (v8.4.1, Bitplane). Chloroplasts (green), peroxisomes (pink), mitochondria (blue), nuclei (yellow) and vacuoles (gray) were reconstructed. After degassing, the cells were exposed to the light treatment.
